# The Results of Intestinal Villi of Laying Hens Exposed With Avian Pathogenic *Escherichia coli* (APEC) After Giving Citric Acid and Dextrose

**DOI:** 10.1155/vmi/6623764

**Published:** 2025-02-24

**Authors:** Sunaryo Hadi Warsito, Mirni Lamid, M. Anam Al-Arif, Herry Agoes Hermadi, Emy Koestanti Sabdoningrum, Siti Rani Ayuti, Yan-Der Hsuuw

**Affiliations:** ^1^Animal Husbandry Division, Faculty of Veterinary Medicine, Universitas Airlangga, Surabaya, East Java, Indonesia; ^2^Veterinary Reproduction Division, Faculty of Veterinary Medicine, Universitas Airlangga, Surabaya, East Java, Indonesia; ^3^Laboratory of Biochemistry, Faculty of Veterinary Medicine, Universitas Syiah Kuala, Banda Aceh, Aceh, Indonesia; ^4^Department of Biological Science and Technology, National Pingtung University of Science and Technology, Pingtung, Taiwan

**Keywords:** avian pathogenic *Escherichia coli*, citric acid, dextrose, intestinal villi, laying hen

## Abstract

The condition known as colibacillosis is still very common in Indonesia, which means that laying hens affected by it are unable to achieve their peak egg production phase. Instead, their egg production is delayed and more susceptible to infection by other diseases. The goal of this study is to determine if the mixture of citric acid and dextrose can inhibit the growth of Avian Pathogenic *Escherichia coli* (APEC) bacteria in laying hens, ultimately leading to the control of colibacillosis cases in Indonesia. A total of 240 laying hen heads in all were split up into 6 treatments, each with 40 replications.The group received the following treatments: T0 is a treatment for laying hens free of APEC infection and they are given no drink that contains any mixture of citric acid and dextrose. T0 (−) is a treatment for laying hens free of APEC infection but a drink mixture of citric acid and dextrose is administered at a dose of 1 g/2.5 L of drinking water. T0 (+) is a treatment for laying hens infected with APEC up to 2 mL/head orally (3 × 10^8^ CFU/mL) and given a drink without the mixture of citric acid and dextrose. T1 is a treatment for laying hens infected with APEC up 2 mL/head orally (3 × 10^8^ CFU/mL) and given a mixture of citric acid and dextrose at a dose of 1 g/1.25 L of drinking water. T2 is a treatment for laying hens infected with APEC up to 2 mL/head orally (3 × 10^8^ CFU/mL) and given a mixture of citric acid and dextrose at a dose of 1 g/2.5 L of drinking water. T3 is a treatment for laying hens infected with APEC up to 2 mL/head orally (3 × 10^8^ CFU/mL) and given a mixture of citric acid and dextrose at a dose of 1 g/3.75 L of drinking water. The results of this study stated that the mixture of citric acid and dextrose showed a significant decrease in the appearance of the intestinal villi of laying hens, because the results were actually less good compared to the treatment infected with APEC. It is suspected that the dose given may still be excessive in concentration. This can be seen from the dose with the highest concentration range (T1) to the lowest (T3) which showed an image of intestinal villi that had a tendency to improve. Therefore, further research and studies are needed regarding the use of a mixture of citric acid and dextrose in laying hens infected with APEC with a lower dose.

## 1. Introduction

The laying hen farming industry in Indonesia is often faced with various challenges, one of which is infection with the Avian Pathogenic *Escherichia coli* (APEC) bacteria. It is known that the results of the multidrug resistance test against APEC in laying hens showed a 62.7% resistance rate [1]. APEC can cause various diseases in laying hens, such as colibacillosis which can result in decreased egg production, poor health, and even death [2, 3]. Overcoming APEC in laying hens requires a comprehensive approach, one of which is the use of feed additives which can help maintain intestinal health. Citric acid is a type of feed additive that has been proven to have benefits in improving the intestinal health of chickens [4]. Meanwhile, dextrose (glucose) is known as an energy source [5]. So, it is very good to give it through drinking water to chickens that are sick, because when feed intake decreases, at least when drinking there is an energy source that can replace it.

The intestine is a vital organ for chickens [6], where nutrients from feed are processed and absorbed. Good intestinal health is very important for: First, optimal nutrient absorption: a healthy intestine can absorb nutrients from feed optimally, so that laying hens get the energy and raw materials needed for growth, egg production, and overall health; Second, strong immunity: a healthy intestine has a strong barrier to prevent pathogenic bacteria [7] such as APEC from entering the body. A healthy gut is also home to beneficial bacteria that help in fighting pathogenic bacteria; Third, high egg production: laying hens with healthy intestines will be more productive in producing eggs [8].

APEC can cause various damages to the intestines of laying hens, including: First, intestinal inflammation: APEC can cause inflammation of the intestines [9], which can interfere with nutrient absorption and increase the risk of other bacterial infections; Second, damage to intestinal villi: Intestinal villi are finger-like structures that help in the absorption of nutrients. APEC can damage the intestinal villi [10], thereby reducing the surface area of the intestine and reducing its ability to absorb nutrients; Third, intestinal cell death: APEC can kill intestinal cells, which can worsen inflammation and disrupt intestinal function.

Citric acid and dextrose have shown potential benefits in improving the intestinal health of laying hens and countering the negative effects of APEC, namely citric acid can help in lowering intestinal pH to a more acidic level, so it is not conducive to the growth of APEC. The administration of citric acid has been proven to be able to lower intestinal pH, so it is natural to reduce pathogenic bacteria [11]. Citric acid can also increase the activity of digestive enzymes and improve immunity [12, 13]. Meanwhile, dextrose can help increase the growth of beneficial bacteria in the intestines and increase the production of short chain fatty acids (SCFA) [14]. For example, the most widely produced SCFA is acetic acid which plays an important role in maintaining intestinal health. Acetic acid is useful in lowering the pH in the intestines, thereby inhibiting the growth of pathogenic bacteria, is the main source of energy for intestinal epithelial cells, and increases the absorption of minerals such as calcium and magnesium [15–17]. Dextrose can also help in increasing blood and oxygen flow to the intestines [18].

This study aims to explain the negative impact of APEC infection on laying hens, introduce citric acid and dextrose as potential feed additives to improve the intestinal health of laying hens, and explain the benefits of citric acid and dextrose on the appearance of the intestinal villi of laying hens exposed to APEC. So, it is hoped that in the end egg production will remain well maintained, if a laying hen farm experiences an APEC bacterial infection. APEC infection in laying hens can cause various negative impacts on intestinal health and egg production. The use of citric acid and dextrose as feed additives has the potential to help improve the intestinal health of laying hens, counteract the negative effects of APEC, and increase egg production. This research is still in the same series as research by [19] which looked at variables in terms of Hen Day Production (HDP) and Feed Conversion Ratio (FCR).

## 2. Materials and Methods

The experimental animals utilized were 26 week old ISA Brown strain laying hens infected with 40 replications and 6 treatments of APEC. Thus, out of a total population of 10,000 heads, 240 heads of laying chickens were used in this investigation. In this study, no power analysis was conducted on the malignancy of APEC bacteria before the study, because the bacterial isolates were obtained directly from the field which had been proven to cause cases of disease. The Laboratory Center for Infection Special Hospitals at Universitas Airlangga was the site of bacterial culture for APEC bacterial isolates, which were acquired from layer farms in the Jabon district of Sidoarjo, East Java, Indonesia. The McFarland I standard was followed in the suspension and dilution of APEC bacteria cultured on EMBA medium (suspension contains 3 × 10^8^ CFU/mL).

The laying hens were initially acclimated to the surroundings for 7 days. Then, on the 8^th^ day, laying hens were orally infected with APECup to 2 mL/head orally (3 × 10^8^ CFU/mL). Over the course of the following 7 days, symptoms included pale, dull-feather chicks that rarely laid eggs and unattractive eggs when they did. According to APEC, the morbidity rate for laying chickens was 100%, whereas the death rate was 0%. Nonetheless, there was a 45%–55% drop in egg output. Subsequently, laying hens received a combination of citric acid and dextrose in their drinking water for 4 weeks, based on the treatment dosage, on the same day.

The group received the following treatments: T0 is a treatment for laying hens free of APEC infection and they are given no drink that contains any mixture of citric acid and dextrose. T0 (−) is a treatment for laying hens free of APEC infection but a drink mixture of citric acid and dextrose is administered at a dose of 1 g/2.5 L of drinking water. T0 (+) is a treatment for laying hens infected with APEC up to 2 mL/head orally (3 × 10^8^ CFU/mL) and given a drink without the mixture of citric acid and dextrose. T1 is a treatment for laying hens infected with APEC up to 2 mL/head orally (3 × 10^8^ CFU/mL) and given a mixture of citric acid and dextrose at a dose of 1 g/1.25 L of drinking water. T2 is a treatment for laying hens infected with APEC up to 2 mL/head orally (3 × 10^8^ CFU/mL) and given a mixture of citric acid and dextrose at a dose of 1 g/2.5 L of drinking water. T3 is a treatment for laying hens infected with APEC up to 2 mL/head orally (3 × 10^8^ CFU/mL) and given a mixture of citric acid and dextrose at a dose of 1 g/3.75 L of drinking water.

Methods for collecting and processing duodenal intestinal samples for histopathology preparation, as follows: First, a 3 cm long section of the small intestine of laying hens is taken and then placed in a small pot containing 10% formalin; Second, fixation is carried out by transferring it to bottle 1 containing 10% formalin buffer for 2 h; Third, dehydration is carried out by placing it in bottle 2 containing 70% alcohol for 1.5 h, in bottle 3 containing 80% alcohol for 1.5 h, in bottle 4 containing 95% alcohol for 1.5 h, in bottle 5 containing absolute alcohol I for 1.5 h, in bottle 6 containing absolute alcohol II for 1.5 h, in bottle 7 containing absolute alcohol III for 1.5 h; Fourth, clearing is done by putting it into bottle 8 containing xylol I for 1 h, into bottle 9 containing xylol II for 1.5 h, into bottle 10 containing xylol III for 1.5 h; Fifth, paraffin infiltration is then done by putting it into bottle 11 containing liquid paraffin I for 1.5 h, into bottle 12 containing liquid paraffin II for 2 h; Sixth, blocking is then done by pouring liquid paraffin into a mold until it solidifies; Seventh, cutting is done with a microtome and further more incubated at a temperature of 50°C for 15 min; The last stage is staining with Hematoxylin–Eosin (HE).

Data on the height of the villi of the small intestine was obtained from the results of making histopath preparations from the duodenum. After microscopic observation of the height mucosa of the villi of the duodenum, each preparation was observed in five randomly selected fields of view and the data obtained was averaged and tabulated carefully [20]. In addition, the study's findings were examined using Analysis of Variance, which was followed by the Duncan's Multiple Range Test.

## 3. Results

**Figure 1:**
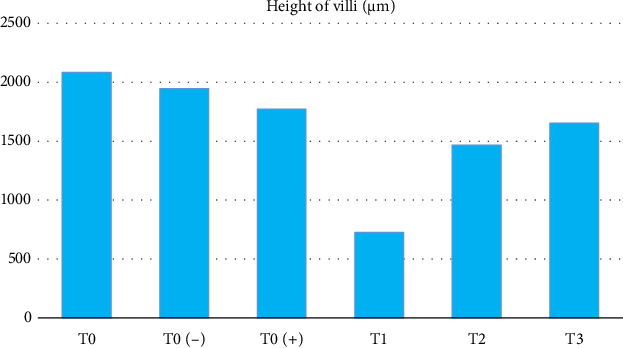
Height of villi.

The results of the study showed that there was a significant difference (*p* < 0.05) in the height of intestinal villi (Table 1 and Figure 1) starting from the highest to the lowest. The order was as follows: T0 as a control group without treatment with an average result of 2084,448 μm, T0 (−) as the control group which was only given a mixture of citric acid and dextrose with an average result of 1945.718 μm, T0 (+) as a group which was only given APEC infection with an average result of 1770,980 μm, T3 as treatment group given APEC infection and given a mixture of citric acid and dextrose at a dose of 1 g/3.75 L of drinking water with an average yield of 1651,826 μm, T2 as the treatment group given APEC infection and given a mixture of citric acid and dextrose with dose of 1 g/2.5 L of drinking water with an average result of 1465.960 μm, T1 as a treatment group given APEC infection and given a mixture of citric acid and dextrose with a dose of 1 g/1.25 L of drinking water with an average result of average 725,454 μm.

**Figure 2:**
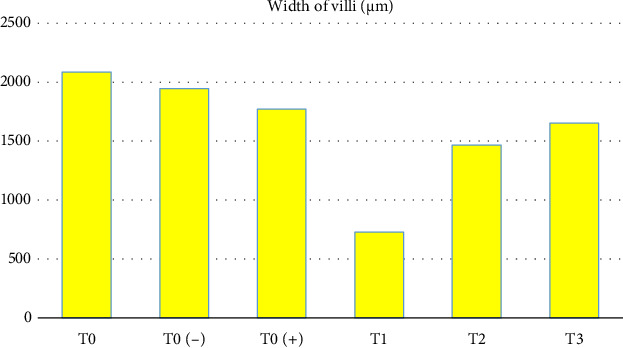
Width of villi.

Likewise, the results of the study showed that there were significant differences (*p* < 0.05) in the width of the intestinal villi (Table 1 and Figure 2), starting from the widest, the sequence was as follows: T0 as the control group without treatment with an average result of 396,944 μm, T0 (−) as a control group which was only given a mixture of citric acid and dextrose with an average yield of 343,696 μm, T0 (+) as a group which was only given APEC infection with an average yield of 331,628 μm, T3 as a treatment group which was given APEC infection and was given the mixture citric acid and dextrose at a dose of 1 g/3.75 L of drinking water with an average result of 313,072 μm, T2 as a treatment group given APEC infection and given a mixture of citric acid and dextrose at a dose of 1 g/2.5 L of drinking water with The average result was 295,74 μm, T1 as the treatment group was given APEC infection and given a mixture of citric acid and dextrose at a dose of 1 g/1.25 L of drinking water with an average result of 313,072 μm.

**Figure 3:**
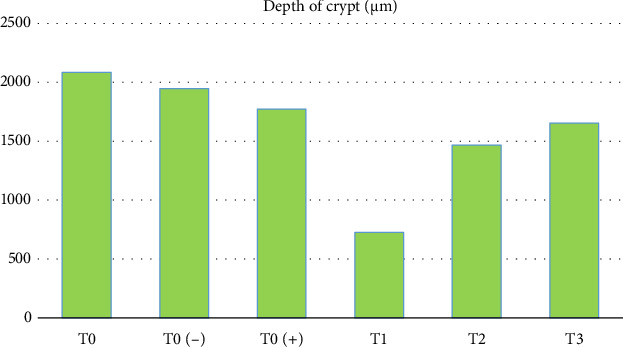
Depth of crypt.

Furthermore, the results of research on crypt depth (Table 1 and Figure 3) also showed that there were significant differences (*p* < 0.05) starting from the highest in order as follows: T0 (−) as the control group which was only given a mixture of citric acid and dextrose with average results-average 324,896 μm, T0 as a control group without treatment with an average result of 317,184 μm, T3 as a treatment group given APEC infection and given a mixture of citric acid and dextrose at a dose of 1 g/3.75 L of drinking water with average results average 312,382 μm, T0 (+) as a group that was only given APEC infection with an average result of 242,992 μm, T2 as a treatment group that was given APEC infection and given a mixture of citric acid and dextrose at a dose of 1 g/2.5 L of drinking water with average yield was 224,008 μm, T1 as the treatment group was given APEC infection and given a mixture of citric acid and dextrose at a dose of 1 g/1.25 L of drinking water with an average yield of 312,382 μm. More complete results of this research can be seen in the table below.

## 4. Discussion

Based on the research data, the results of the intestinal villi images were quite interesting. Because in general, the administration of a combination of citric acid and dextrose to laying hens exposed to APEC bacteria, the results of the intestinal villi images were actually not as good as the treatment exposed to APEC without being given a combination of citric acid and dextrose or T0 (+) treatment. The results of the intestinal villi images on the height of the villi, the width of the villi and the lowest crypt depth were at T1 with results (725,454 μm; 222,786 μm; 217,504 μm) at a combination dose of citric acid and dextrose of 1 g/1.25 L of drinking water or the dose with the highest concentration. Then at a dose with a medium concentration or 1 g/2.5 L of drinking water, the results improved better (1465,960 μm; 295.474 μm; 224,008 μm). Furthermore, at the lowest concentration dose or 1 g/3.75 L of drinking water, the results increased even better than the dose with medium concentration (1651,826 μm; 313,072 μm; 312,382 μm). These results are close to the T0 (+) treatment, even in the width of the villi the results are no longer significantly different (*p* > 0.05). This fact indicates a strong suspicion that the dose given for the combination of citric acid and dextrose is still relatively high in concentration. Because as it is known, citric acid has a negative effect, namely, being an irritant [21, 22]. This can be seen on examination of many intestinal villi that are destroyed, especially at high concentrations. So, if it look at the results of this study, it appears that there is a tendency for the height of the villi, the width of the villi and the depth of the crypts to decrease along with increasing concentrations of the mixture of citric acid and dextrose.

Meanwhile, according to [19], which is a series of this research, shows the results that HDP and FCR actually give the same results (*p* > 0.05) as healthy conditions (controls that were not given any treatment), which means they perform better than those exposed to APEC (*p* < 0.05). But there is a tendency for better results along with the decrease in the dose of the mixture of citric acid and dextrose, although not significantly different (*p* > 0.05). As obtained data, the results of HDP from a high concentration dose (1 g/1.25 L of drinking water) to low (1 g/3.75 L of drinking water) are 90.75%; 95.25%; 98.25%. While in the control group that was not exposed to APEC and was also not given a mixture of citric acid and dextrose, the HDP results were 96.85%. For the group exposed to APEC and not given a mixture of citric acid and dextrose, the HDP results were 65.75%. Furthermore, in the results of the FCR, the results of the study from a high concentration dose (1 g/1.25 L of drinking water) to low (1 g/3.75 L of drinking water) were 1.75; 1.63; 1.63. Meanwhile, in the control group that was not exposed to APEC and was also not given a mixture of citric acid and dextrose, the FCR result was 1.77. For the group exposed to APEC and not given a mixture of citric acid and dextrose, the FCR result was 2.17. So, based on the data from the results of the HDP and FCR studies, it further strengthens the suspicion that the dose of the mixture of citric acid and dextrose used in this study is still too high. Although the HDP and FCR results are as good as the control, based on the description of the intestinal villi, it is still not good because it is still below the group exposed to APEC without being given a mixture of citric acid and dextrose. Therefore, further research is needed to determine the most appropriate dose (a mixture of citric acid and dextrose concentrations to be further reduced), so that the HDP and FCR results remain good or even better but the description of the intestinal villi is at least the same as normal conditions.

When combined, both conditions, namely by administering a mixture of citric acid and dextrose which is strongly suspected of still being at a high concentration used for laying hens exposed to APEC bacteria, the results obtained are poor intestinal villi but the HDP and FCR results are actually better. This condition is suspected to occur because: First, citric acid is indeed effective in inhibiting the growth of APEC bacteria, but it can also affect the condition of the intestinal villi. This occurs because too much acid can damage the structure of the intestinal villi; Second, dextrose as an energy source can help laying hens exposed to APEC bacteria recover faster from infection. So, that this additional energy can be allocated to egg production; Third, chickens have a compensation mechanism to maintain egg production. This means that even though nutrient absorption is disrupted due to damage to the intestinal villi, chickens can still utilize existing energy reserves to produce eggs.

So, the possibility that what happened in this study was caused by the role of citric acid, as an acidifier, has several benefits in counteracting the negative effects of APEC on the intestines of laying hens, namely: lowering intestinal pH: citric acid can reduce intestinal pH to a more acidic level (pH 3.3–4.0) [23], so it is not conducive to APEC growth. APEC prefers neutral or alkaline intestinal conditions [24]; increases digestive enzyme activity: The acidic conditions created by citric acid can increase the activity of digestive enzymes, such as protease and amylase [25, 26], which help laying hens digest feed and absorb nutrients; promote the growth of intestinal villi: citric acid can promote the growth of intestinal villi [27, 28], which are important structures in nutrient absorption. Healthy and long intestinal villi help laying hens absorb more nutrients from feed, thereby increasing health and egg production; improves immunity: citric acid can improve the immunity [29] of laying hens by increasing the production of white blood cells and increasing antibody activity. This helps laying hens fight APEC infections.

Dextrose, as an energy source, also has several benefits in counteracting the negative effects of APEC on the intestines of laying hens, namely: increasing the growth of beneficial bacteria: dextrose can be a source of energy for beneficial bacteria in the intestine [30], such as *Lactobacillus* and *Bifidobacterium*. These beneficial bacteria help in controlling the APEC population and maintaining gut health; increases production of SCFA: dextrose can be fermented by beneficial bacteria in the intestine to produce SCFA [31]. SCFA have various benefits for intestinal health, such as increasing nutrient absorption, strengthening the intestinal barrier, and improving immunity [32, 33]; improves the health of intestinal villi: dextrose can help in improving the health of intestinal villi by increasing the flow of blood and oxygen to the intestines [34]. This helps the intestinal villi absorb nutrients more optimally.

The mixture of citric acid and dextrose can affect the composition of the intestinal microflora in a complex way. At low concentrations, both can promote the growth of beneficial bacteria that can help digestion and absorption of nutrients, which in turn increases HDP and decreases FCR [19]. However, at high concentrations, both can also suppress beneficial bacteria, causing an imbalance in the intestinal microflora and negatively affecting villus height, villus width, and crypt depth.

On the other hand, it is possible that the mixture of citric acid and dextrose can have antagonistic effects on the intestinal villi. At low concentrations, the positive effects may be more dominant. Citric acid can increase mucus secretion, which can protect the villi from damage caused by APEC infection. Because citric acid has acidic properties, it can stimulate mucus production as the body's natural mechanism to protect the lining of the digestive tract from acidity [35]. On the other hand, dextrose can increase villous cell proliferation [36], which can help regenerate damaged villi. This ultimately results in a better picture of the intestinal villi. However, at high concentrations, the antagonistic effects can cancel each other out, or even the negative effects of dextrose can dominate, resulting in a lower picture of the intestinal villi. These differences in mechanisms of action may explain why the effects on intestinal villi are not always unidirectional.

A mixture of citric acid and dextrose may be more effective in counteracting the negative effects of APEC on the intestines of laying hens than either ingredient alone. Because citric acid helps create an environment that is not conducive to APEC growth, while dextrose helps promote the growth of beneficial bacteria and the production of SCFAs [14]. This combination may help in: First, improving intestinal health: a healthy intestine is more resistant to APEC infection and is better able to absorb nutrients; Second, increasing egg production [19]: laying hens with a healthy intestine will be more productive in producing eggs; Third, improving egg quality: eggs produced by laying hens with a healthy intestine will be of better quality, with higher protein and vitamin content.

Chicken's response to a mixture of citric acid and dextrose may vary depending on individual factors, such as age, health, and genetics. This may explain why some chickens show increases in villi height, villi width, and crypt depth, whereas others do not. It is important to note that this research is still in its early stages and requires further research to fully understand the mechanisms behind the observed effects. Some additional studies that may help clarify these findings include: First, more detailed analysis of gut microflora to identify specific bacteria affected by citric acid and dextrose; Second, studies on a wider range of citric acid and dextrose concentrations to determine the optimal dose range for positive effects on intestinal villi; Third, research on different APEC strains to see if the effects vary depending on the type of bacteria. With further research, it is hoped that scientists can better understand how a mixture of citric acid and dextrose can be used to improve gut health and performance in laying hens, especially in the context of APEC infections.

## 5. Conclusions

The mixture of citric acid and dextrose did not show better benefits on the appearance of the intestinal villi of laying hens, because the results were actually less good compared to the treatment exposed to APEC. However, based on this series of studies in terms of HDP and FCR, it actually gave the same results as chickens in healthy condition (without treatment) or the results were better than those exposed to APEC. It is suspected that the dose given was still excessive in concentration. This can be seen from the dose with the highest concentration range (T1) to the lowest (T3) which showed an image of intestinal villi that had a tendency to improve. Therefore, further research and studies are needed regarding the use of a mixture of citric acid and dextrose in laying hens infected with APEC with a lower dose. So, that the use of a mixture of citric acid and dextrose as a feed additive can be one of the useful strategies to improve the health and productivity of laying hens, especially in conditions that are at high risk of APEC infection.

## Figures and Tables

**Table 1:** Description of the height of the villi, width of the villi, and depth of crypts from the intestine duodenum in laying hens infected with APEC.

Treatments	Height of villi ± SE (μm)	Width of villi ± SE (μm)	Depth of crypt ± SE (μm)
T0	2084.448^f^ ± 39.567	396.944^d^ ± 15.470	317.184^d^ ± 9.550
T0 (−)	1945.718^e^ ± 33.740	343.696^e^ ± 17.238	324.896^e^ ± 12.433
T0 (+)	1770.980^d^ ± 29.984	331.628^c^ ± 12.674	242.992^c^ ± 10.271
T1	725.454^a^ ± 25.405	222.786^a^ ± 10.972	217.504^a^ ± 7.326
T2	1465.960^b^ ± 28.268	295.474^b^ ± 13.341	224.008^b^ ± 8.578
T3	1651.826^c^ ± 30.652	313.072^bc^ ± 11.268	312.382^d^ ± 9.693

*Note:* Different superscripts indicate significant differences (*p* < 0.05).

## Data Availability

The data supporting this study are not publicly available due to privacy or ethical restrictions.

## Nomenclature

APEC: Avian pathogenic *Escherichia coli*
SE: Standard error
T0: Control
T0 (−): Negative control
T0 (+): Positive control
T1: First treatment
T2: Second treatment
T3: Third treatment
EMBA: Eosin methylene blue agar
CFU: Colony forming unit
SCFA: Short chain fatty acid
HDP: Hen day production
FCR: Feed conversion ratio
